# Standardization via Post Column Infusion—A Novel and Convenient Quantification Approach for LC-MS/MS

**DOI:** 10.3390/molecules29163829

**Published:** 2024-08-12

**Authors:** Katharina Habler, Arber Rexhaj, Felix L. Happich, Michael Vogeser

**Affiliations:** Institute of Laboratory Medicine, LMU University Hospital, LMU Munich, Marchioninistr. 15, 81377 Munich, Germany

**Keywords:** post column infusion, isotope-labeled internal standard, quantification techniques, liquid chromatography tandem mass spectrometry

## Abstract

Mass spectrometry (MS) is a widely used analytical technique including medical diagnostics, forensic toxicology, food and water analysis. The gold standard for quantifying compounds involves using stable isotope-labeled internal standards (SIL-IS). However, when these standards are not commercially available, are prohibitively expensive, or are extremely difficult to synthesize, alternative external quantification techniques are employed. We hereby present a novel, convenient and cheap quantification approach—quantification via post column infusion (PCI). As a proof of concept, we demonstrated PCI quantification for the immunosuppressant tacrolimus in whole blood using liquid chromatography–tandem mass spectrometry (LC-MS/MS). The validation results met the criteria according to the guideline on bioanalytical method validation of the European Medicine Agency (EMA), achieving imprecisions and inaccuracies with coefficient of variation and relative bias below 15%. Anonymized and leftover whole blood samples from immunosuppressed patients receiving tacrolimus were used for method comparison (PCI quantification vs. conventional internal standard (IS) quantification). Both methods showed strong agreement with a Pearson correlation coefficient of r = 0.9532. This novel PCI quantification technique (using the target analyte itself) expands the quantification options available in MS, providing reliable results, particularly when internal standards are unavailable or unaffordable. With the current paper, we aim to demonstrate that our innovative PCI technique has great potential to overcome practical issues in quantification and to provide guidance on how to incorporate PCI in existing or new LC-MS methods. Moreover, this study demonstrated a more convenient method for correcting matrix effects in comparison to alternative PCI techniques.

## 1. Introduction

Mass spectrometry (MS) is a widely used analytical technique to identify and/or quantify a variety of molecules by their mass-to-charge ratio. Tandem mass spectrometry (MS/MS) coupled with gas chromatography (GC-MS/MS) or especially with high-performance liquid chromatography (HPLC-MS/MS) is applicable across diverse fields including medical diagnostics, forensic toxicology, environmental, food and water analysis. For targeted approaches, precise quantification is of utmost importance [1,2,3,4].

In the middle of the 20th century, the term “stable isotope dilution analysis” (SIDA) was first described in the literature in the context of the quantification of amino acids and fatty acids. The first modern SIDA, i.e., the quantification of a compound via its stable isotope labeled internal standard (SIL-IS), was published in 1966 for the quantification of glucose via sevenfold deuterated glucose as SIL-IS as trimethylsilyl derivatives using GC-MS [5,6,7].

Since then, accurate quantification via SIL-IS has been considered the gold standard procedure in the field of MS. However, the lack of SIL-IS availability for many natural products and pharmaceuticals is a key problem in the application of this technique. In residue analysis (particularly for pesticides) and in clinical laboratories, analogues, homologues or derivatives of the analyte are used as internal standards (ISs) as an alternative to SIL-IS for standard routine applications, but also for candidate reference measurement procedures (RMP) based on LC-MS/MS, e.g., therapeutic drug monitoring (TDM) of vancomycin (glycopeptide antibiotic) or RMP of gentamicin (aminoglycoside antibiotic) [8,9]. Moreover, due to the lack of pure and high-quality SIL-IS for tacrolimus (immunosuppressant), the ethyl analog ascomycin has been and is still partially used today as an IS for quantification and TDM in clinical routine diagnostics [10].

In addition to quantification by IS or SIL-IS in LC-MS/MS analysis, there are also external quantification procedures that are used, i.a., in residue, environmental, water or food analysis, e.g., standard addition, solvent or matrix-matched calibration, and ECHO technique: A possible alternative for quantification, such as the standard addition method, in which several aliquots of the extract are fortified with increasing amounts of the analyte of interest, does not require any IS, but is very time-consuming and requires a linear response of the analytes. Moreover, the analyte concentration in the sample must be estimated as precisely as possible in a first run. The complex handling and the multiple injections often make this method impractical for routine analyses, particularly when dealing with a large number of samples. In contrast, quantification by means of external matrix-matched calibration, for which no IS is required, involves preparing calibration standards with the target analytes in a sample matrix that is similar or identical to the actual sample being analyzed. This procedure provides more reliable results with regard to matrix effects for low or moderately loaded samples with a uniform matrix. It must also be ensured that the blank material is free of the target analytes, which can be a problem, especially with endogenous substances. Otherwise, a solvent calibration is often used, the suitability of which must be checked with regard to ion suppression and ion enhancement. Another quantification method without SIL-IS is the ECHO technique. In this procedure, the non-labeled target compound serves as the IS. The sample and IS solution are injected one after the other with a short delay during a chromatographic run. This delay can also be created by an additional precolumn. The analyte from the sample and the IS (ECHO of the analyte) elute with a slight difference in retention time. This approach requires baseline separation of the analyte peak and the ECHO peak in such a way that coeluting matrix components and other analytes either do not affect both signals or affect them identically [11,12,13,14,15,16].

As early as 1999, Choi et al. proposed the use of a post column infusion (PCI) as a means of correcting for ion suppression on both the drug and its earlier eluting metabolites [17]. Despite the innovative quantification technique, its implementation in LC-MS methods was infrequent. The restricted implementation of PCIs may be attributed to an inadequate recognition of the issue of matrix effects and a reluctance to abandon the widely recognized method for matrix effect correction (i.e., SIDA). In addition, PCI analysis and its calculation of concentrations with the existing vendors’ software is complex, as it is mainly designed for SIDA in MS/MS. 

Rossmann et al. and Liao et al. demonstrated that their PCI-based approaches can be incorporated into the analysis of complicated samples with complex matrices, offering a valuable alternative to the conventional IS methodology [18,19,20].

What these publications have in common is that either the SIL-IS of the target analyte(s) or a structural analogue to the target analyte(s) is infused. PCI quantification has so far mainly been described for insecticides in wheat hay, pharmaceuticals in urine and amino acids in plasma [17,18,19,20].

The aim of our work is to present a novel and convenient quantification approach for LC-MS/MS—quantification via PCI (using the target analyte itself) based on the US patent “Functional check and variance compensation in mass spectrometry” [21]—as a recent addition to extend quantification possibilities applicable in every area of MS and in highly complex sample matrices, especially when IS and/or SIL-IS are unavailable or non-affordable.

As proof of concept for PCI quantification, we chose tacrolimus in whole blood as the analyte, which is a commonly administered immunosuppressant for which we have been offering TDM in routine clinical practice for more than two decades.

## 2. Results

### 2.1. Proof of Concept—Quantification via PCI

**Figure 1:**
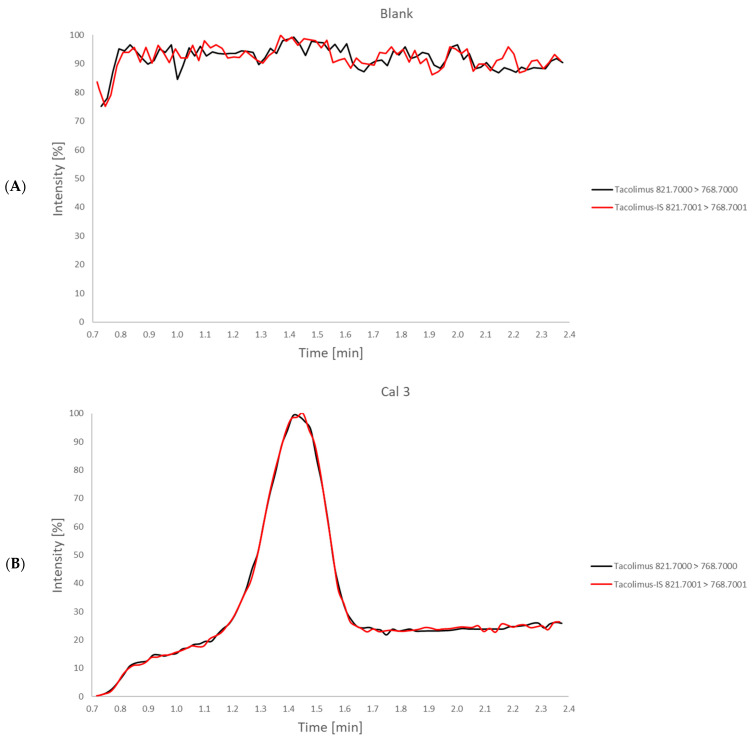

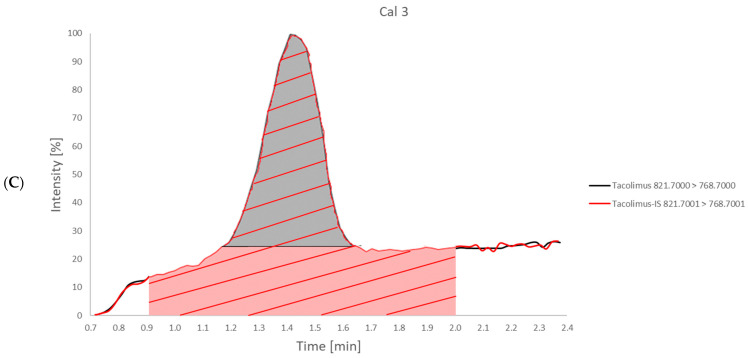
Post column infusion (PCI)-LC-MS/MS chromatograms of (**A**) a blank sample. A continuous baseline signal of the target analyte (here tacrolimus) is generated by PCI of a solution of the target analyte. Two mass transitions traces for the same analyte are acquired in parallel (tacrolimus 821.7000 > 768.7000 in black and tacrolimus-IS 821.7001 > 768.7001 in red). (**B**) calibrator 3 (11.6 ng/mL) with tacrolimus (black) and tacrolimus-IS (red). The calibrators, quality controls (QCs), and unknown samples are injected into the LC-MS/MS-system, resulting in two (but identical) peak signals for tacrolimus (black) and tacrolimus-IS (red) after chromatographic separation. (**C**) integration of area tacrolimus (grey), area tacrolimus-IS (red hatched) and area IS (externally infused tacrolimus, light red). Automatic software peak integration is applied for tacrolimus (black), leading to the area tacrolimus (grey area). Manual peak integration for a fixed elution time window (here from 0.9 to 2.0 min) is used for tacrolimus-IS (red), leading to area tacrolimus-IS (red hatched area). The actual area of the internal standard (IS) (light red area) that represents the externally infused tacrolimus is calculated by area tacrolimus-IS (red hatched area)—area tacrolimus (grey area).

As a proof of concept and as a new and convenient quantification approach for LC-MS/MS quantification via PCI, the analyte (here tacrolimus) was consistently post column-infused into the MS during each run by an integrated syringe pump. The externally infused analyte tacrolimus served as the internal standard (IS) for quantification. This created a continuously higher baseline throughout the measurement (Figure 1A). For ease of calculation and quantification, the second multiple reaction monitoring (MRM) for tacrolimus was adjusted in the fourth decimal digit and named ‘tacrolimus-IS’ (Table 1). Due to the resolution of the MS/MS, tacrolimus (821.7000 > 768.7000) and tacrolimus-IS (821.7001 > 768.7001) can be considered identical, but two different mass traces were recorded in the software. 

Figure 1A,B show the recorded MRMs (tacrolimus in black and tacrolimus-IS in red) in a blank sample and in calibrator 3, respectively. Through a combination of automatic software peak integration for tacrolimus (grey peak area) and manual integration for a fixed elution time window from 0.9 to 2.0 min for tacrolimus-IS (red hatched peak area), all required metadata could be exported from the MS software (v 4.1) and imported into Excel. The actual area IS (light red peak area) that represents the externally infused tacrolimus is calculated by area tacrolimus-IS (red hatched peak area)—area tacrolimus (grey peak area)—as demonstrated in Figure 1C (see Equation (1)).

**Figure 2:**
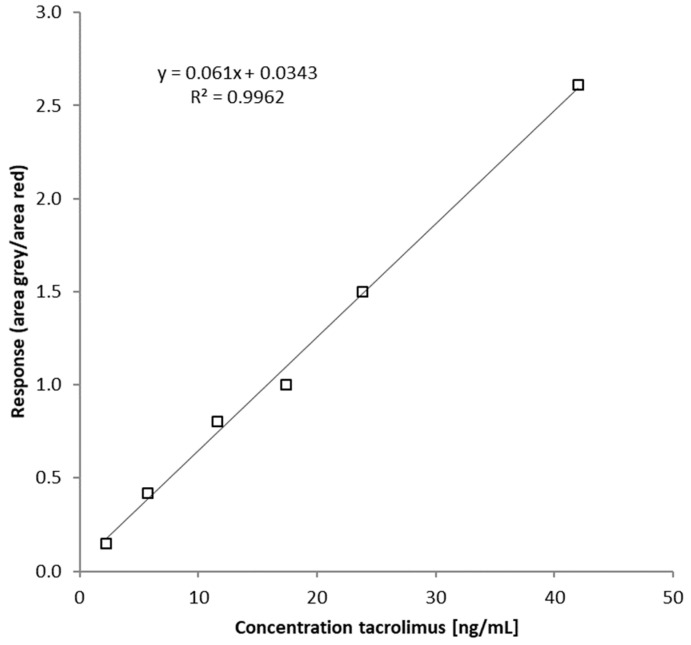
Calibration curve with 6 calibration points (squares): response (area tacrolimus (grey)/area IS (externally infused tacrolimus, light red)) is plotted against concentration of tacrolimus.

The response is derived from the ratio of the area of the tacrolimus (grey) divided through the area of the IS (light red, see Equation (2). The externally infused tacrolimus serves as the IS. This response is used to generate a calibration function (see Equation (3)) to quantify quality controls (QCs) and unknown samples in the same way as done with conventional internal standardization (Figure 2).

### 2.2. Performance Criteria

All investigated analytical performance aspects of the quantification of tacrolimus levels via PCI (externally infused tacrolimus) fulfilled the criteria of the guideline on bioanalytical method validation of the European Medicine Agency (EMA) [22]. 

In all measurement series, the coefficient of determination for the respective linear regression was between 0.9670 and 0.9962, indicating a high linear relationship over the whole calibration range. Calibratior 1 with 2.22 ng/mL tacrolimus was set as the lower limit of quantification (LLOQ) and calibrator 6 with 42.0 ng/mL tacrolimus as the upper limit of quantification (ULOQ). In all analyses, no carry-over was observed for either tacrolimus or ascomycin. For all measurements, the area of the calculated area IS did not differ by more than 8.27%, regardless of blank, calibrator, QC, patient sample, and tacrolimus concentration. This consistency of the IS area (externally infused tacrolimus) showed no significant matrix effects, which are essential for quantification via PCI. For this novel quantification concept (externally infused analyte tacrolimus), intra- and inter-day inaccuracies and imprecisions for all 4 QCs ranged from −14.8% to 2.75% and from 2.53% to 12.5%, respectively (Table 2), and they were within the required ±15% of the guideline on bioanalytical method validation of EMA (nominal concentration to calculated concentration) [22].

### 2.3. Method Comparison

**Figure 3:**
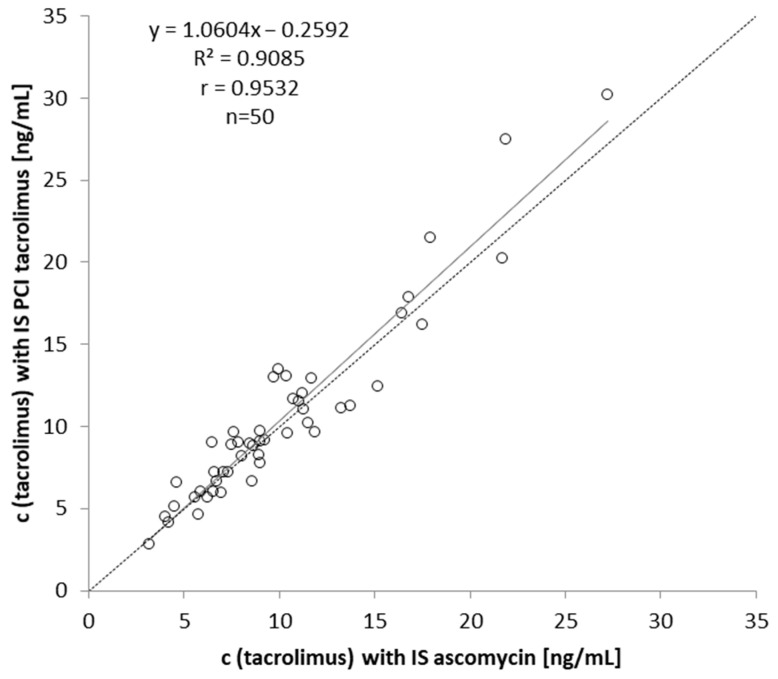
Method comparison of tacrolimus levels (*n* = 50) quantified by PCI of tacrolimus versus ascomycin as IS.

In total, 50 leftover and anonymized whole blood samples from immunosuppressed patients receiving tacrolimus were quantified by two different methods: (1) PCI quantification: externally infused tacrolimus was used as the IS, and the quantification was performed as described above. (2) The conventional quantification was applied with ascomycin as the IS. Figure 3 shows the results of the method comparison. The range of the tacrolimus levels was from 3.16 to 27.2 ng/mL for PCI as the quantification approach (mean at 10.1 ng/mL, median at 9.00 ng/mL) and from 2.85 to 30.2 ng/mL for ascomycin as the conventional IS (mean at 10.5 ng/mL, median at 9.12 ng/mL). All patient values were within the calibration range. Both quantification approaches showed strong agreement, with a Pearson correlation coefficient of r = 0.9532.

## 3. Discussion

With PCI quantification (using the target analyte itself), we have introduced a new and convenient quantification approach in LC-MS/MS analysis. We used tacrolimus, a frequently administered immunosuppressant, as a proof of concept and successfully validated this quantification approach according to the guideline on bioanalytical method validation of EMA, and we tested it on authentic whole blood samples [22]. Intra- and inter-day imprecisions and inaccuracies were independent of the concentrations of the QCs below ±15%. The IS area (see Equation (1); externally infused tacrolimus by the syringe pump, which resulted in a higher required baseline) differed by no more than 9%. This consistency showed no significant matrix effects independent on the calibrator, QC, and sample injected, as potential interferences can reach the ion source and MS together with the target substance and the infused substance. Due to identical physical and chemical properties and in order to better compensate for any ion suppression or enhancement, we used the same substance for PCI as the target substance in our approach. Other published approaches for PCI quantification rely on SIL-IS or ISs as externally infused components, with a long list of requirements to be met for the successful selection of a PCI-IS candidate [23]. Additionally, due to the use of an IS or, in particular, SIL-IS, the problem of an expensive or barely available IS persists.

The method comparison, PCI quantification vs. conventional quantification via IS ascomycin, with 50 anonymized and leftover authentic whole blood samples showed very good agreement with a Pearson’s r of 0.9532. Although PCI quantification cannot compensate for losses during sample preparation, we could demonstrate that even highly complex matrices such as whole blood can be reliably analyzed and quantified with this method. However, a standardized protocol including automatic software peak integration and fixed manual peak integration (here form 0.9–2.0 min for tacrolimus-IS), processing, and calculation is a fundamental requirement for obtaining consistent, reliable, and comparable results. Our proof-of-concept study on PCI quantification with the target analyte itself for quantification was applied to one analyte, tacrolimus, in whole blood, but is not limited to single-component analysis and can also be extended to a multi-analyte method if required.

PCI quantification is a competitive method compared to quantification methods such as standard addition, solvent or matrix-matched calibration, and the ECHO technique. Despite the high costs of SIL-IS assays, they remain the gold standard quantification procedure in LC-MS/MS, as SIL-IS can compensate for both matrix effects and analyte losses during sample preparation and thus provide highly reliable results [24]. However, PCI quantification using the target analyte for quantification is a simple, convenient, and cost-effective method for the quantification of substances for which no IS is commercially available or where the IS is very challenging to synthesize.

Particularly in multi-component analysis, it is often difficult to find a suitable IS or SIL-IS for each analyte, which would also greatly increase the cost of the analysis and make the method with several hundred additional mass transitions more challenging. Multi-residue analysis is often applied in foods for the simultaneous analysis of a large number of veterinary drugs (e.g., antimicrobials, antimycotics, sedatives, and on-steroidal anti-inflammatory drugs) in animal products or pesticide residues in fruits, vegetables, cereals, meat, fish, dairy, and processed products. In this field of MS analysis, quantification is therefore routinely performed using matrix-matched calibration and standard addition methods [11,14,25,26].

In clinical routine diagnostics and in toxicological residue analysis, quantification using ISs or SIL-IS is relied upon whenever possible.

The lack of SIL-IS availability is also particularly relevant for new or rare target substances, such as residues, drugs, or respective metabolites. In the future, PCI quantification, which uses the target analyte itself for quantification, may also be a way to compensate for matrix effects and to achieve reliable results in (multi-residue) food, drug, and omic analysis.

## 4. Materials and Methods

### 4.1. Chemicals and Reagents

Water, acetonitrile, methanol, and ammonium acetate (ULC/MS—CC/SFC grade) were obtained from Biosolve (Valkenswaard, The Netherlands). Zinc sulfate heptahydrate was obtained from Carl Roth (Karlsruhe, Germany). Calibrators and QCs (part of the kit “MassTox^®^ Immunosuppressants in Whole Blood—LC-MS/MS”) were purchased from Chromsystems (Gräfelfing, Germany). The analyte tacrolimus and IS ascomycin were obtained from Sigma-Aldrich (St. Louis, MO, USA).

### 4.2. Calibrators, Quality Controls, Internal Standards, Precipitation Reagent, and Post Column Infusion Solution

The commercial kit “MassTox^®^ Immunosuppressants in Whole Blood—LC-MS/MS” (Chromsystems) includes 1 blank, 6 calibrators, and 4 QCs. According to the product information leaflet, all lyophilized calibrators and QCs were reconstituted in water. The concentrations of all levels are given in Table 3. Stock solutions of the analyte tacrolimus and the IS ascomycin were prepared in methanol at a concentration of 50 µg/mL and 100 µg/mL, respectively. The precipitation reagent consisted of 1% zinc sulfate in water/methanol (22/78; *v*/*v*) containing ascomycin as IS with 9.8 ng/mL. The PCI solution had a concentration of 0.15 ng/mL tacrolimus in 2 mM ammonium acetate in water/methanol (1/9; *v*/*v*), which also comprised mobile phase B. Calibrators, QCs, stock solutions, precipitation reagent, and PCI solution were aliquoted and stably stored at −20 °C.

### 4.3. Patient Samples

In total, 50 leftover and anonymized EDTA whole blood samples from immunosuppressed patients receiving tacrolimus were analyzed and used for method comparison. The samples were stably stored at 8 °C for no longer than 2 days until analysis.

### 4.4. Sample Preparation

For sample preparation, samples (blank, calibrator, QC, patient whole blood samples) were thoroughly homogenized by overhead mixing, and 150 µL of each sample was added to 150 µL precipitation reagent in a 2 mL safe lock tube. After vortexing for 10 s and mixing for 5 min by means of a horizontal shaker, the samples were centrifuged at ambient temperature for 10 min at 16,000 g. The supernatant was transferred in a glass vial with insert.

### 4.5. LC and MS Parameters

The LC-MS/MS system consisted of an Acquity UHPLC with autosampler, a binary pump, a switching valve, a syringe pump, and a column oven coupled with a triple quadrupole mass spectrometer Xevo TQ-S (Waters, Milford, MA, USA). The MassLynx V 4.1 software (Waters) was used.

The weak wash and strong wash solvents were water/methanol (9/1; *v*/*v*) and acetonitrile, respectively. The mobile phases consisted of water (A) and 2 mM ammonium acetate in water/methanol (1/9, *v*/*v*) (B), with an isocratic elution set at 7% A and 93% B and a flow rate of 0.4 mL/min. The autosampler and column manager were tempered to 8 °C and 45 °C, respectively. For chromatographic separation, a BEH C18 column (100 × 2.1 mm, 1.7 µm, Waters) was used. Four µL of the samples were injected, while the PCI solution was infused directly into the MS via an integrated syringe pump at a flow rate of 20 µL/min (3 pg/min). The switching valve in combined mode (0.6–2.4 min) allowed elution entry into the MS of both the binary pump and the syringe pump. From 0 to 0.6 min and from 2.4 to 3.0 min, the flow of the binary pump was directed into the waste and the syringe pump was switched off. After the total run time of 3 min, the syringe pump was automatically refilled with PCI solution.

MS/MS measurement was performed with positive electrospray ionization (ESI+). The optimal parameters were determined by direct infusion of tacrolimus and ascomycin: source temperature 150 °C, desolvation temperature 500 °C, desolvation gas flow 800 L/Hr, cone gas flow 150 L/Hr, capillary voltage 1.5 kV, cone voltage 20 V. As precursor ions, the [M + NH_4_]^+^ adducts were measured. All measured precursor and product ions with collision energies (CEs) and retention times of tacrolimus (as analyte), tacrolimus used as IS (tacrolimus-IS), and ascomycin are given in Table 1.

### 4.6. Proof of Concept—Quantification via PCI

For the quantification via PCI, the respective analyte (here tacrolimus) had to be delivered by the integrated syringe pump during each run, which resulted in a continuously higher baseline. According to Table 1, two different mass traces were recorded—mass transition 821.7000 > 768.7000 for tacrolimus (Figure 1, red) and mass transition 821.7001 > 768.7001 for tacrolimus-IS (Figure 1, black). In the processing software (TargetLynx (v 4.1), Waters), tacrolimus was defined as analyte and tacrolimus-IS was defined as IS. The peak of tacrolimus (821.7000 > 768.7000) with a retention time of 1.43 min was on the top of the higher baseline and was integrated automatically by the software. The signal of tacrolimus-IS (821.7001 > 768.7001) required accurate manual integration from 0.9 to 2.0 min, including higher baseline and peak, which ultimately corresponded to the combined areas of analyte tacrolimus in, e.g., calibrator, QC, or sample and externally infused tacrolimus. Integrated peak areas were exported from TargetLynx and imported into Microsoft Excel^®^, which was used for further calculations. For all measurements, the difference between area tacrolimus-IS (Figure 1C, red hatched) and area tacrolimus (Figure 1C, grey) was calculated to finally obtain the actual area of externally infused tacrolimus (area IS, Figure 1C, light red).

Area (IS) = Area (tacrolimus-IS) − Area (tacrolimus)(1)

The response was calculated as follows:

Response = Area (tacrolimus)/Area (IS)(2)

A calibration curve was plotted with response against concentration, and the respective linear equation was calculated, as follows:

y = m × x + t(3)

This function was used for further calculations of tacrolimus levels in calibrators, QCs, and unknown samples. For comparing the individual tacrolimus levels, quantification of tacrolimus was also performed by the conventionally used IS ascomycin via TargetLynx.

### 4.7. Performance Criteria

The analytical performance of this novel quantification approach via PCI of the respective analyte was validated in terms of linearity, carry-over, matrix effect, inaccuracy, and imprecision of QCs. All validation and performance aspects were compared to the requirements of the guideline on bioanalytical method validation of EMA [22].

For addressing the linearity of every calibration series, the linear equation, the coefficient of determination (R2), and the intercept were considered. Carry-over was determined by the area of a blank sample (without native tacrolimus) injected after the highest calibrator (calibrator 6, upper limit of quantification, ULOQ). This area should not exceed 20% of the area of the lowest calibrator (calibrator 1, lower limit of quantification, LLOQ). The carry-over for ascomycin was also checked, for which the area in a blank sample should be less than 5%. The consistency of the area of the IS (externally supplied tacrolimus) over each measurement series independent of the injected sample (calibrator, QC, patient sample with different levels of tacrolimus) was monitored to check for matrix effects such as ion enhancement or ion suppression. Intra-day inaccuracy and imprecision were tested by replicate analysis of all 4 QCs (*n* = 5). Inter-day inaccuracy and imprecision were evaluated by replicate analysis of all 4 QCs over 3 independent measurement series (*n* = 3, 3 days). Imprecision was expressed as coefficient of variation (CV) and inaccuracy as relative bias. According to the guideline on bioanalytical method validation of EMA, both should be within ±15%. [22]

### 4.8. Method Comparison

As a preliminary experiment, 50 leftover and anonymized whole blood samples from immunosuppressed patients receiving tacrolimus were analyzed for method comparison. The tacrolimus levels were quantified by tacrolimus infused as PCI solution (PCI quantification) and by ascomycin as conventional IS. Both values were compared to evaluate quantification via PCI.

## 5. Conclusions

We have described here a novel and more convenient quantification approach in LC-MS/MS analyses’ quantification via PCI that uses the target analyte itself for quantification. In our proof-of-concept study, PCI quantification of tacrolimus in authentic whole blood samples provided reliable results comparable to the conventional IS quantification. However, an exact protocol for peak integration and data processing is a fundamental requirement for a successful application. In retrospect, and especially when SIL-IS are not available, this PCI quantification approach can provide a tool to compensate for matrix effects and to achieve reliable results in food, drug, and “omic” analysis. 

## 6. Patents

United States Patent Geyer et al., “Functional check and variance compensation in mass spectrometry”. Patent No. US 8,829,429 B2 [21].

## Figures and Tables

**Table 1:** MS parameters of measurands.

Measurand	Precursor Ion, m/z	Product Ion, m/z	CE, V	Retention Time, min
Tacrolimus	821.7000	768.7000	18	1.43
Tacolimus-IS	821.7001	768.7001	18	1.43
Ascomycin	809.7000	756.7000	20	1.43

**Table 2:** Intra-day (*n* = 5) and inter-day (*n* = 3) inaccuracies and imprecisions of all 4 QCs quantified by PCI.

QC	Concentration, ng/mL	Intra-Day (*n* = 5) Inaccuracy, %	Intra-Day (*n* = 5) Imprecision, %	Inter-Day (*n* = 3) Inaccuracy, %	Inter-Day (*n* = 3) Imprecision, %
QC I	2.75	−14.8	2.53	2.75	12.5
QCII	7.51	−10.1	4.78	−14.7	9.56
QC III	15.7	−5.70	2.22	−12.1	9.84
QC IV	33.2	−12.1	5.01	−13.9	7.65

**Table 3:** Concentration of calibrators and QCs of tacrolimus.

Calibrator/QC	Concentration Tacrolimus, ng/mL
Blank	-
Calibrator 1	2.22
Calibrator 2	5.75
Calibrator 3	11.6
Calibrator 4	17.4
Calibrator 5	23.9
Calibrator 6	42.0
QC I	2.75
QC II	7.51
QC III	15.7
QC IV	33.2

## Data Availability

The original contributions presented in this study are included in this article; further inquiries can be directed to the corresponding author.
